# An enhanced round robin using dynamic time quantum for real-time asymmetric burst length processes in cloud computing environment

**DOI:** 10.1371/journal.pone.0304517

**Published:** 2024-08-15

**Authors:** Most. Fatematuz Zohora, Fahiba Farhin, M. Shamim Kaiser

**Affiliations:** 1 Computer Science and Engineering, Bangladesh Army International University of Science and Technology, Cumilla, Bangladesh; 2 Computer Science and Engineering, International University of Business Agriculture and Technology, Dhaka, Bangladesh; 3 Institute of Information Technology, Jahangirnagar University, Dhaka, Bangladesh; The Islamia University of Bahawalpur Pakistan, PAKISTAN

## Abstract

Cloud computing is a popular, flexible, scalable, and cost-effective technology in the modern world that provides on-demand services dynamically. The dynamic execution of user requests and resource-sharing facilities require proper task scheduling among the available virtual machines, which is a significant issue and plays a crucial role in developing an optimal cloud computing environment. Round Robin is a prevalent scheduling algorithm for fair distribution of resources with a balanced contribution in minimized response time and turnaround time. This paper introduced a new enhanced round-robin approach for task scheduling in cloud computing systems. The proposed algorithm generates and keeps updating a dynamic quantum time for process execution, considering the available number of process in the system and their burst length. Since our method dynamically runs processes, it is appropriate for a real-time environment like cloud computing. The notable part of this approach is the capability of scheduling tasks with asymmetric distribution of burst time, avoiding the convoy effect. The experimental result indicates that the proposed algorithm has outperformed the existing improved round-robin task scheduling approaches in terms of minimized average waiting time, average turnaround time, and number of context switches. Comparing the method against five other enhanced round robin approaches, it reduced average waiting times by 15.77% and context switching by 20.68% on average. After executing the experiment and comparative study, it can be concluded that the proposed enhanced round-robin scheduling algorithm is optimal, acceptable, and relatively better suited for cloud computing environments.

## Introduction

Cloud Computing (CC) has become a popular technology in the modern world due to its cost savings and the scalable, accessible, and flexible services it provides for businesses and individuals [1]. This technology is aimed at reducing operational costs by providing on-demand resources such as data storage, physical and virtual servers, applications, development tools, and network capabilities via the internet [2, 3]. These resources are hosted and maintained by a remote Cloud Services Provider (CSP) that controls the user access to each resource using specific scheduling algorithms [4]. The number of resources determines the degree of simultaneous execution by a CSP. Several task scheduling algorithms such as First Come First Serve (FCFS), Shortest Job Fast (SJF), Longest Job First (LJF), Feedback Based Task Scheduling (FBTS), Priority-Based (PB), and Round Robin (RR) are incorporated to fairly and efficiently distribute these resources against each user requests [5–9]. The objective of these scheduling algorithms is to maximize resource utilization, enhance the quality of service, minimize waiting times, turnaround times, and response times, reduce costs, manage complex tasks, and ensure adaptability in a dynamic CC environment [10]. To meet this objective, researchers have proposed various scheduling algorithms considering the system’s requirements.

**Fig 1:**
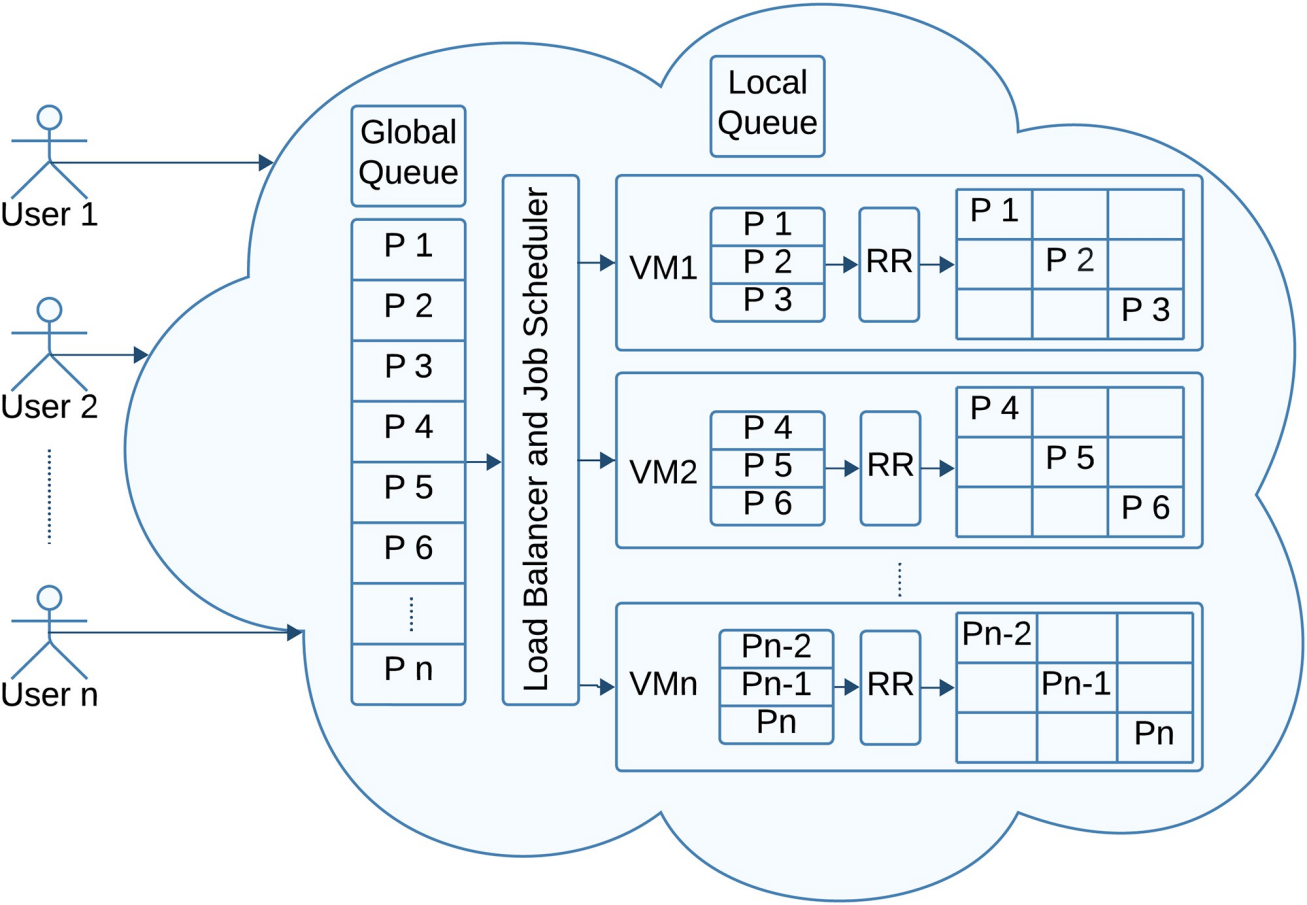
Round robin scheduling in cloud computing environment.

RR is a popular scheduling approach with a fixed amount of CPU slot called Quantum Time (QT) for each process in the ready queue to execute them fairly without leaving any process waiting infinitely [11, 12]. Fig 1 displays the CC environment that uses the RR scheduling approach to execute user tasks simultaneously. The remote user sends workload requests like data processing, computation, or any other operation to execute in the cloud environment. A job scheduler manages the allocation of resources and schedules the execution of user requests from the global queue in the distributed infrastructure. Each CSP has several virtual machines (VMs), virtualized instances of a physical computer capable of running applications and operating systems. A load balancer is employed to circulate traffic across multiple VMs equally to optimize resource utilization and ensure that no single server is overwhelmed with too much workload. VM executes user requests through multi-programming to minimize the total execution time and incorporates different scheduling approaches. Understanding the dynamic nature of virtual machines (VMs) within CC environments is crucial for efficient task scheduling, ensuring optimal resource allocation and system performance [13]. Fig 1 shows that the VMs are using the RR algorithm to complete the tasks in a cyclic order.

The main challenge of RR is the selection of the optimal QT [14]. A small QT results in a higher Context Switching (CS) leading to additional overhead and reduced CPU efficiency whereas a large QT may increase the Average Waiting Time (AWT) of the system. The rule of thumb for selecting the optimal QT is 80 percent of the process bursts should be shorter than the QT [15]. But processes in CC arrive in the system at different times with variable length Burst Time (BT). As a result, selecting a fixed QT using this rule at the beginning may become irrelevant later. To address this issue our paper proposed a dynamic time quantum based RR approach that can manage processes with variable length BT and enhance the overall system performance reducing the AWT and unnecessary CS in the CC environment. The suggested approach improved and concentrated on a number of crucial aspects of scheduling techniques, including:

- Our technique dynamically executes processes, which makes the approach suitable for a real-time setting such as CC
- Automatically tear down the convoy effect caused by processes with relatively larger burst length and adjust the optimal QT for the existing processes in the system
- Provides a relatively small AWT, ATT, and an even distribution of context switches throughout the execution of processes

The complete proposed algorithm has been described in the methodology section with an appropriate flowchart. We have tested the proposed method on three distinct datasets: process BT in ascending order, descending order, and random order considering 20 processes for each test case. Our dataset has skewness in the data with nonuniform process BT which is a common scenario in CC. The algorithm determines QT each time a new process arrives in or leaves the system ensuring that the QT calculation considers the remaining process only. The objective of this work is to minimize the AWT and number of CS which are the crucial performance factor in the scheduling. Comparing the proposed method with some other existing improved RR algorithms it is clear that our algorithm is capable of meeting the objective for nonuniform and realistic data.

The rest of the paper is structured as follows: In section two we discussed some improved RR approaches in recent years including their outcomes and limitations. Section three describes the methodology of the proposed Enhanced Round Robin with Dynamic Time Quantum (ERRDTQ) approach with algorithm and flowchart. This section also includes the dataset we considered to evaluate our work. Experimental results and necessary discussion with appropriate tables and charts have been demonstrated in section four. Finally, the conclusion and future direction of this research is presented in section five.

## Related work

Omotehinwa et al. presented a dynamic CPU scheduling algorithm (SIDRR) that uses a numeric outlier detection technique and geometric mean to determine an optimal time quantum for processes with asymmetrically distributed burst times. They implemented and tested the proposed algorithm alongside existing improved variants of Round-Robin (RR) scheduling algorithms [16–20]. For that, they feed the processes used in each paper as input into the programs written in C programming language for each of the selected algorithms to validate the correct implementation. Experimental analysis has been done based on the arrival time of processes, considering zero arrival time and non-zero arrival time categories, and different orders of burst time of processes (ascending, descending, and random order). Then they compared the performance of the proposed algorithm with the selected improved variants of RR in terms of average waiting time, average turnaround time, and number of context switches. The proposed algorithm outperformed the other variants regarding average waiting time and average turnaround time, except for EDRR, which performed better in context switching [21].

Alhaidari et al. proposed a novel technique focusing on the traditional RR algorithm disadvantages. The proposed model optimizes the functionality of the traditional RR algorithm for scheduling tasks in the cloud computing environment by optimizing the performance metrics by decreasing the average waiting time, average turnaround time, and average response time. The proposed technique is called the dynamic round-robin heuristic algorithm (DRRHA) which considers the mean of time quantum and remaining burst time of tasks. It focuses on addressing the time quantum issue by computing the average for all the tasks in the ready queue, which is organized based on the shortest job first (SJF) approach. They dynamically adjust the time quantum by dividing the calculated average by the current process’s BT or remaining BT which is repeated for each task and each iteration. Additionally, it is crucial to employ the principle of checking the remaining burst time of the tasks. If the remaining burst time is less than or equal to the current task quantum, the task execution is finalized and subsequently removed from the ready queue. Otherwise, the task is stored at the end of the ready queue to be executed in the subsequent iteration. However, their algorithm calculates relatively lower QT for the larger BT in the queue creating unnecessary context switches. Numerous experiments were conducted using the CloudSim Plus tool to assess the DRRHA and then compared with related proposed algorithms [17, 22–25]. They found it outperforms other studied algorithms in terms of performance metrics like average waiting time, average turnaround time and average response time [26].

Sakshi et al. propose the Median-Average Round Robin (MARR) scheduling algorithm, which dynamically adjusts the time quantum to improve the performance compared to static Round Robin scheduling and other existing dynamic scheduling techniques. The authors compare the proposed MARR algorithm with four other scheduling algorithms to demonstrate its effectiveness [27–32]. The paper highlights the impact of the quantum decision on the scheduling of processes and the system’s performance, emphasizing the need for an improved algorithm. The MARR algorithm incorporates meta-heuristic optimization strategies, making it a better-performing algorithm in scheduling techniques. The algorithm that has been proposed, along with all the performance metrics, offers the most optimal outcomes for Average Turnaround Time (ATT) and Average Waiting Time (AWT). However, the context switches remain relatively constant across all dynamic scheduling algorithms that have been considered [33].

In recent times, more research has been done based on the optimization of round-robin algorithms in cloud computing environments [34–37]. Dipto et al. proposed a new round-robin task scheduling approach named NRRTSA that enhances the performance of the allocation of resources from the central remote server in a cloud computing environment. As well as the implemented NRRTSA has also determined an efficient time quantum for each round during scheduling. They determine the time quantum dynamically based on the differences among the three maximum burst times of tasks in the ready queue for each round. It utilizes an additive manner among the differences and the burst times of the processes while determining the time quantum. By reducing average turn-around time, diminishing average waiting time, and minimizing the number of context switching, it outperforms other existing round-robin task scheduling approaches for the cloud computing environment [38].

Another study has been conducted by Nermeen et al. proposing the utilization of the ameliorated round-robin algorithm (ARRA) as a means of task scheduling in cloud computing. This algorithm has demonstrated that an ideal time quantum of (0.75 times the average) should be allocated to the tasks, with an increasing order of priority. The algorithm was then simulated and compared against other algorithms such as RR, Improved RR, Enhanced RR, ARR, and Enhanced RR (RAST ERR) [34, 39–41]. The experimental results have indicated that the ARRA algorithm has significantly reduced the Average Waiting Time (AWT) by 3.8-38.20% and the Average Turnaround Time (ATT) by 2.28-38.19% in comparison to the other algorithms [42].

Task scheduling and allocation algorithms have been thoroughly studied in recent years in cloud computing research, taking into account both single and multi-objective optimization viewpoints. In order to improve system efficiency, single-objective optimization seeks to minimize metrics like makespan (response time). Numerous studies have used novel methodologies, such as discrete Particle Swarm Optimization (PSO), hybrid Genetic Algorithm (GA), and Simulated Annealing (SA) techniques [43, 44]. In contrast, multi-objective optimization involves simultaneous consideration of multiple criteria, such as makespan minimization and monetary cost reduction [45–47]. Researchers have delved into the intricacies of trade-offs inherent in workflow scheduling to address these diverse objectives.

Although the goal of this study is to improve RR scheduling for asymmetric burst length processes in real-time in cloud computing environments, it is important to recognize the wider field of scheduling algorithms and their goals. This work attempts to integrate traditional scheduling algorithms into the cloud computing setting, in contrast to many previous research that mostly concentrate on cloud-specific solutions. Therefore, this study has not explored the single-point and multi-point optimization perspectives. However, understanding the multidimensional nature of scheduling algorithms and their implications for broader goals is critical for further study in this area.

A summary of the highlighted work done in this field is given in Table 1.

**Table 1:** Summary of related studies.

Ref	Contributions	Time Quantum	Dataset Consideration	Limitations
[16]	Proposed an improved RR scheduling with minimizes AWT and ATT using arithmatic mean of the processes BT	QT = BT or Mean	5 processes	BT of the processes are considered as symmetric statistical distribution patterns.
[18]	This paper presents a variant of RR scheduling algorithm using dynamic QT to improve the performances	QT = Mean	5 processes in 3 different order	Processes are assumed to have BT within a specific range
[20]	Developed an efficient RR using the thumb rule of selecting QT more than 80% Processes’ BT	QT = 0.8 * Maximum_BT	5 processes	Outlier BT is not considered while executing
[21]	Improved the AWT and ATT of scheduling with burst times that are asymmetrically distributed using Interquartile Range(IQR) method for outlier detection	QT = BT or Third Quartile or Arithmatic Mean or (Arrithmatic Mean + First Quartile/2)	20 processes in 3 different orders	The IQR method may fail to detect outliers in small datasets due to limited data and overidentify outliers in large datasets due to natural variability.
[23]	Proposed a variation of the RR technique that might be applied to situations where the processes’ initial BT are unknown. At run time, the time quantum must be adjusted for this	QT = random number or (QT*2) or (QT/2)	5 processes	A process with a short burst time could enter in the middle of execution when the quantum is increased, which could cause the algorithm to suffer because the new process would have to wait longer than the original RR.
[26]	This paper dynamically adjusts the QT in the RR algorithm based on the mean and remaining burst time of tasks, improving task execution continuity and scheduling efficiency	QT = (Mean/2) + ((Mean/2)/BT)	5 processes in 3 different order BT range from 10 to 90	The datasets under consideration are uniformly distributed and homogeneous. For smaller processes, this approach produces significantly bigger QT, and for larger processes, smaller QT, which is irrelevant.
[32]	Developed an improved RR model using dynamic QT to gain better average response time and AWT than the classical RR	QT = (median * highest BT)	6 processes	The median rule is unable to manage processes with too short BT or too large BT.
[33]	Used a median-average based approach to set the QT dynamically for effective scheduling	QT = (Median + Mean) / 2	8 processes in 3 different order	Convoy effect results from an inability to identify the skewnesses in the BT of the processes.
[38]	The suggested method improved performance using the three highest BT of the processes in the ready queue to dynamically determine the time quantum for each round	QT = (Highest BT—1) or Highest BT	4 datasets each having 5 distinct processes	Performance degradation for processes arrived with BT in descending order.
[42]	Designed a dynamic QT to enhance overall scheduling performance based on the arithmetic mean of the process length.	QT = (3/4 * Mean)	8 processes in 3 different order	Unable to effectively manage the diversity in process BT.

## Methodology

The proposed approach in this research works on minimizing the ATT and AWT using dynamic QT which is updated each time a new process arrives in the system or a process leaves the system terminating its execution. The QT is determined considering the available burst time of all the processes in the ready queue. The QT will be updated in a manner so that 80 percent of the ready processes can complete their execution in a single turn. To facilitate the small processes earlier for maximizing the overall performance the remaining burst time of a running process will also be tracked in several checkpoints and the scheduler will decide context switching based on the remaining BT and current QT.

### Definitions

This subsection presents the abbreviation used in the algorithm. Let,

*P*_*i*_ = *i*^*th*^ number process,

*AT*_*i*_ = Arrival Time of *i*^*th*^ number process,

*BT*_*i*_ = Burst Time of *i*^*th*^ number process,

RQ = Ready Queue,

SRQ = Sorted Ready Queue,

QT = Quantum Time,

RBT = Remaining Burst Time of running process *P*_*i*_,

FBT = Burst Time of the First process in the Sorted Ready Queue.

### Proposed algorithm

In our proposed algorithm [Algorithm 1], Enhanced Round Robin with Dynamic Time Quantum (ERRDTQ), allows processes to join the system in real-time and add them to the ready queue, which is ordered in ascending order by process burst time. A new time quantum is also computed with the 80^th^ percentile formula. The approach functions preemptively, applying the computed time quantum to the first process in the sorted ready queue until a new process joins the queue. The amount of burst time that the running process has left determines whether to interrupt it. The process can conclude its execution if the burst time left is less than one-third of the current time quantum. On the other hand, if this threshold is exceeded by the remaining burst duration, the algorithm looks for an alternative process in the ready queue with a burst time less than one-third of the current time quantum and assigns CPU to that process preempting the running process. The preempted process is then reintegrated into the ready queue with its updated burst time.

**Fig 2:**
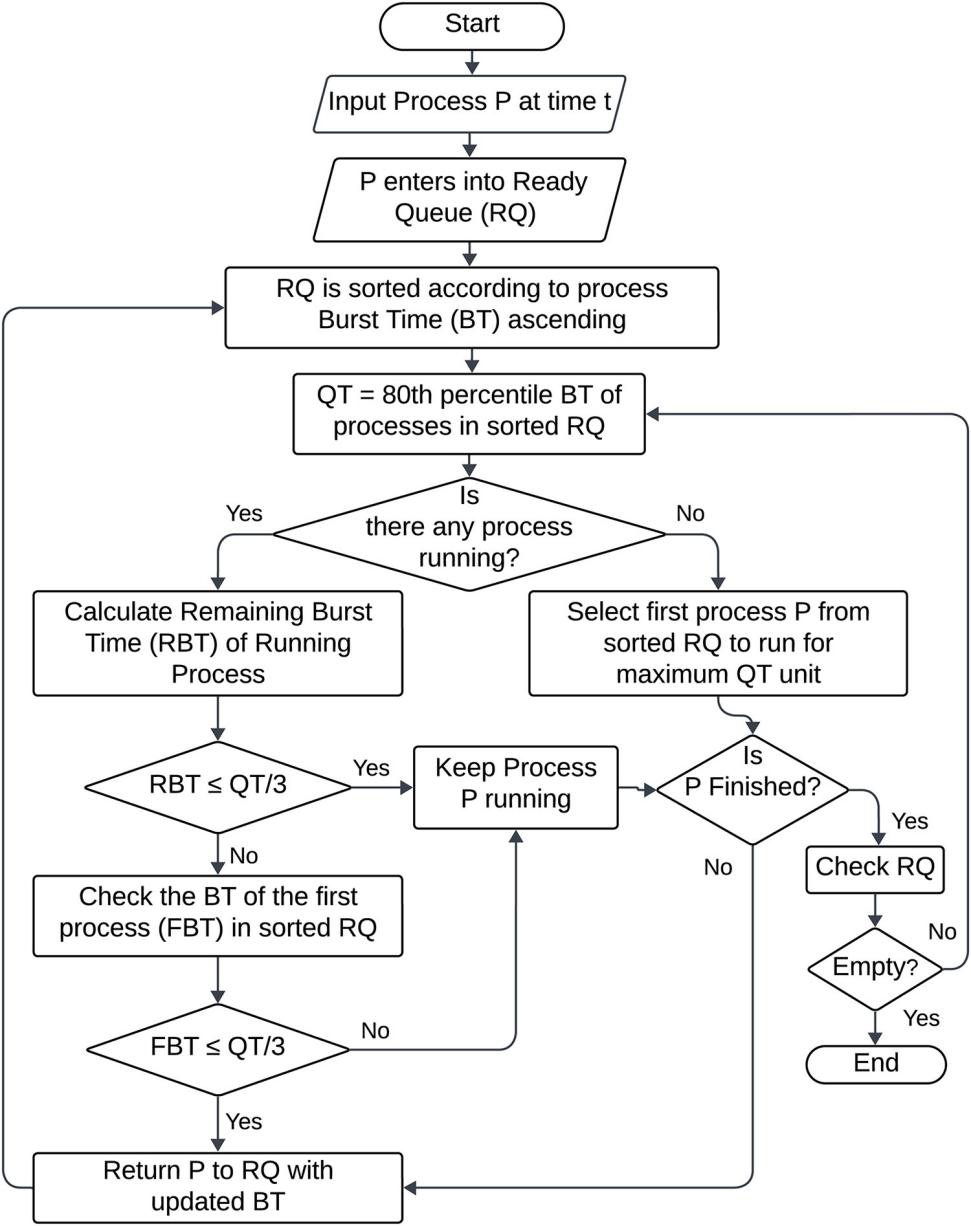
Flow chart of ERRDTQ.

In this algorithm, the arrival or termination of a process works as a checkpoint for the scheduler. The design incorporates maximum 2n numbers of time quantum corresponding to n enqueue and dequeue operation, ensuring that processes with the shortest burst times are executed first, thereby minimizing average waiting time. Simultaneously, the algorithm strategically preempts running processes, taking into account their remaining burst times, thereby contributing to a balanced approach to context switching. Fig 2 depicted the detailed flow chart of our proposed algorithm.

**Algorithm 1** ERRDTQ

1: Initialize new Process *P*_*i*_ at *AT*_*i*_ with *BT*_*i*_

2: Process *P*_*i*_ enters into the RQ

3: *SRQ* ← *Sort*(*RQ*)       ▷ according to the BT in Ascending order

4: *N* ← *Length*(*SRQ*)

5: (*QT*) ← *SRQ*[0.8 * *N*]

6: **if** CPU is empty **then**

7:  Select first process P from SRQ to execute for maximum QT unit

8:  Go to step 24

9: **else**

10:  Calculate RBT of running Process P

11:  **if** RBT ≤ QT/3 **then**

12:   Keep executing P for QT unit

13:   Go to step 24

14:  **else**

15:   Check the FBT in the SRQ

16:   **if** FBT ≤ QT/3 **then**

17:    Return P to RQ with updated BT

18:   **else**

19:    Keep executing P for QT unit

20:    Go to step 24

21:   **end if**

22:  **end if**

23: **end if**

24: **if** P is finished **then**

25:  Check SRQ

26:  **if** SRQ is empty **then**

27:   End

28:  **else**

29:   Go to step 5

30:  **end if**

31: **else**

32:  Return P to RQ with updated BT

33: **end if**

### Quantum Time (QT) calculation

The 80^th^ percentile value displayed in [Algorithm 2] is used by the suggested ERRDTQ technique to ascertain the optimum time quantum for task scheduling. Let’s look at an example that will assist in grasping this method better. Assume the following 12 values make up our dataset: [12, 45, 67, 23, 89, 34, 56, 78, 90, 10, 350, 101]. This dataset can be sorted to provide the following results: [10, 12, 23, 34, 45, 56, 67, 78, 89, 90, 101, 350]. We multiply 0.8 by 12 to find the index for the 80th percentile, and the result is 9.6. We obtain 10 by rounding this to the closest whole number. Consequently, our desired Quantum Time is represented by the 10^th^ number of the sorted dataset, which in this case is 90.

**Algorithm 2** Quantum Time Calculation

1: *n* ← number of processes in SRQ

2: *index* ← 0.8 * *n*

3: **if** index is an integer **then**

4:  go to step 8

5: **else**

6:  *index* ← nearest whole number

7: **end if**

8: *QT* ← *SRQ*[*index*]

### Experimental data

The processes’ arrival times convey the basis of the experimental analysis. The processes’ burst times in ascending [Table 2], descending [Table 3], and random [Table 4] order were considered. We evaluated twenty processes with different arrival and burst times. The skewness of the data was taken into consideration while generating the process’s burst time. The analysis was carried out under the assumptions of a single processor environment, considering burst times before execution, and non-consequential sorting time.

**Table 2:** Processes with burst time in ascending order.

Process ID	A	B	C	D	E	F	G	H	I	J	K	L	M	N	O	P	Q	R	S	T
**Arrival Time**	0	1	2	3	4	5	6	7	8	9	10	11	12	13	14	15	16	17	18	19
**Burst Time**	10	15	34	37	37	44	46	51	52	55	68	71	72	74	77	79	88	91	101	350

**Table 3:** Processes with burst time in descending order.

Process ID	A	B	C	D	E	F	G	H	I	J	K	L	M	N	O	P	Q	R	S	T
**Arrival Time**	0	1	2	3	4	5	6	7	8	9	10	11	12	13	14	15	16	17	18	19
**Burst Time**	350	101	91	88	79	77	74	72	71	68	55	52	51	46	44	37	37	34	15	10

**Table 4:** Processes with burst time in random order.

Process ID	A	B	C	D	E	F	G	H	I	J	K	L	M	N	O	P	Q	R	S	T
**Arrival Time**	0	1	2	3	4	5	6	7	8	9	10	11	12	13	14	15	16	17	18	19
**Burst Time**	51	77	44	10	79	34	88	68	72	74	15	55	91	37	71	101	350	52	37	46

## Results and discussion

This section demonstrates the optimal process execution by the proposed ERRDTQ approach with different datasets, and evaluates and compares the experiment result with five existing improved round-robin algorithms. The proposed method has shown comparatively outstanding performance for any kind of dataset to minimize the waiting time of the processes, balance the context switching and give a response to a waiting process in a productive manner.

### Performance metrics for CPU scheduling algorithm

Numerous performance metrics are used in CPU scheduling to evaluate the efficiency of scheduling algorithms. Context switching, average waiting time, and average turnaround time are a few of the important performance indicators. To measure our proposed scheduling algorithms’ effectiveness and ensure that processes are executed promptly and efficiently, these indicators are employed in this experiment.

#### Context switching

The technique of switching the CPU from one process to another while ensuring that the previous process’s information is preserved and can be resumed later is known as context switching. In the multi-programming environment, context switching is initiated to ensure the maximum throughput of the processor. The OS can invoke context switching if a running process is waiting for an I/O or synchronization action to complete, an interrupt occurs, a transition between the user mode and kernel mode is required or a process’s time quantum expires. However, context switching comes at a cost, for example, Performance overhead, Cache and Translation Lookaside Buffer (TLB) flushes, loss of energy, confusion about priorities, and even a decline in cognitive function. While designing a scheduling algorithm it is preferred to minimize the frequency of context switching as well as ensure a balanced response to the available processes.

#### Average Turnaround Time (ATT)

The whole amount of time a process spends in the system to finish its job, including any waiting in the ready queue, is known as turnaround time. The calculation involves determining the difference between each process’s arrival and termination times, then averaging these numbers. Smaller ATT indicates that processes are executing quickly.

$$\begin{array}{c} ATT=\frac{\sum_{i=1}^{n}\left(Completion_{i}-Arrival_{i}\right)}{n} \end{array}$$

#### Average Waiting Time (AWT)

A significant metric in CPU scheduling is the AWT, indicating the amount of time a process spends in the ready queue for completing its execution. It is determined as the difference between a process’s turnaround and burst time. The CPU scheduling procedure and process arrival sequence have a direct influence on the average waiting time. One of the primary objectives of scheduling algorithms is to reduce the overall waiting time.

$$\begin{array}{c} AWT=\frac{\sum_{i=1}^{n}\left(Turnaround_{i}-Burst_{i}\right)}{n} \end{array}$$

### Experimental results

The overall process of the proposed ERRDTQ has been mathematically explained in this subsection. The determination of the effective time quantum for the proposed approach and the execution of all tasks with the determined effective time quantum have been described here. Intel Core i7 processor with 16 GB of RAM and 4GB Nvidia 920MX GPU were used in the preliminary test. The experiment was carried out using 3 types of datasets shown in [Tables 2–4] all having a nonzero arrival time and variable length burst time.

#### Case 1: Burst time in ascending order

**Fig 3:**
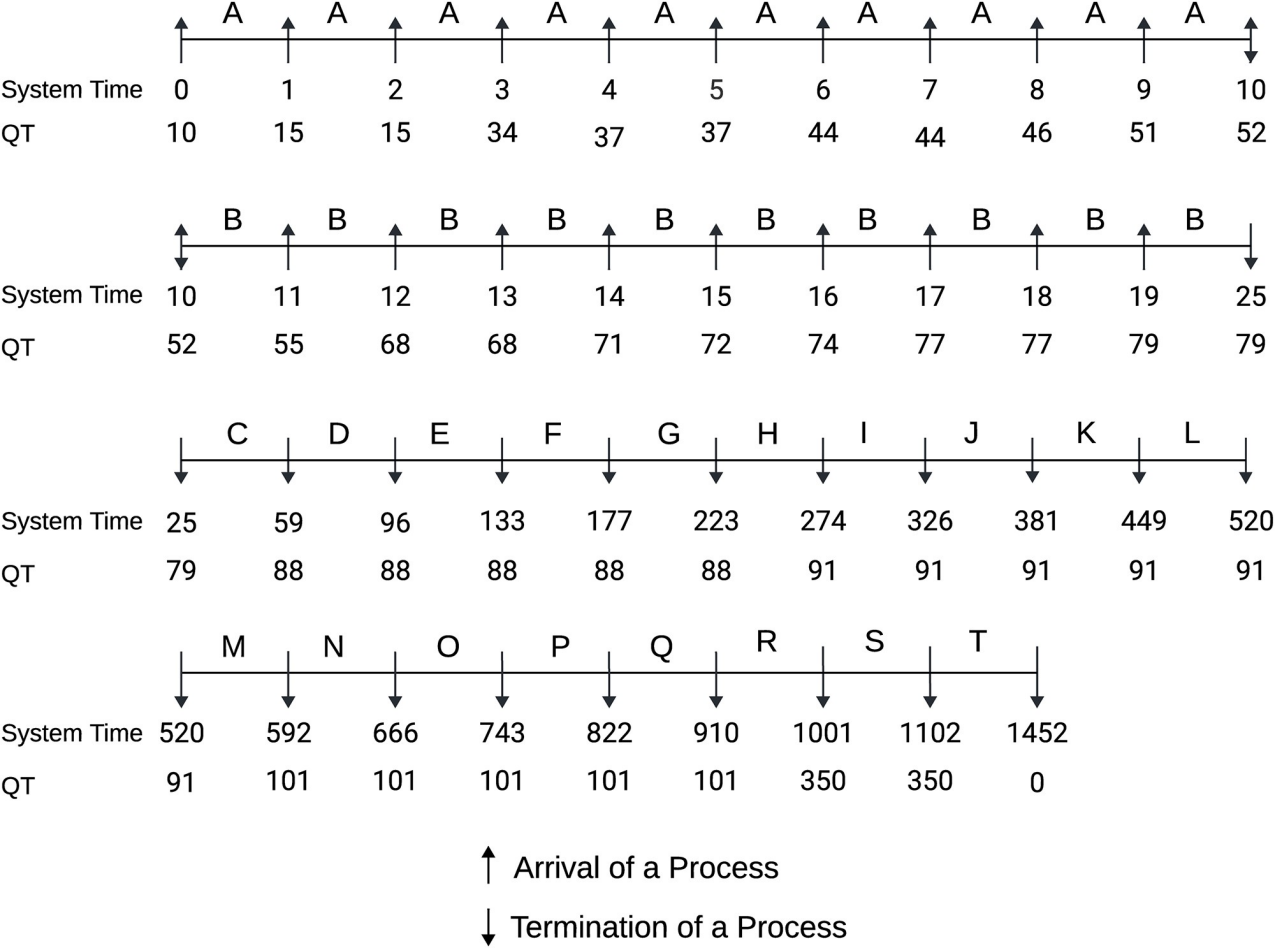
Gantt chart of the process with burst time in ascending order.

Here, all the processes are assumed to arrive at the system with their burst time in an increasing manner. Our algorithm is designed to calculate the QT in a dynamic method with an enqueue or dequeue operation in the SRQ. Fig 3 depicted the Gantt chart of the process execution with determined QT at every checkpoint. At time 0, process A has arrived in the RQ with BT = 10 and is selected by the scheduler for execution. While executing A, 10 other processes B, C, D, E, F, G, H, I, J, and K have enqueued in the RQ at time 1, 2, 3, 4, 5, 6, 7, 8, 9, 10 respectively. Based on their BT, all the incoming processes are sorted in ascending order and the algorithm determines a new QT each time a new process arrives. As the remaining BT of the running process (A) is smaller than one-third of the current QT, other processes are kept waiting in the SRQ. At time 10 process A left the system and process B is selected for execution as it has the smallest BT among all the available processes in SRQ and occupied the CPU till 25. In the meantime, we received 9 other processes L, M, N, O, P, Q, R, S, and T in the system at time 11, 12, 13, 14, 15, 16, 17, 18, 19 respectively. At time 25, we have 18 processes in the SRQ ready for the CPU. The scheduler selects a process with the minimum BT and executes it until no interruption occurs. The proposed ERRDTQ completed all 20 tasks with 19 context switches, 415.95 AWT and 488.55 ATT.

#### Case 2: Burst time in descending order

**Fig 4:**
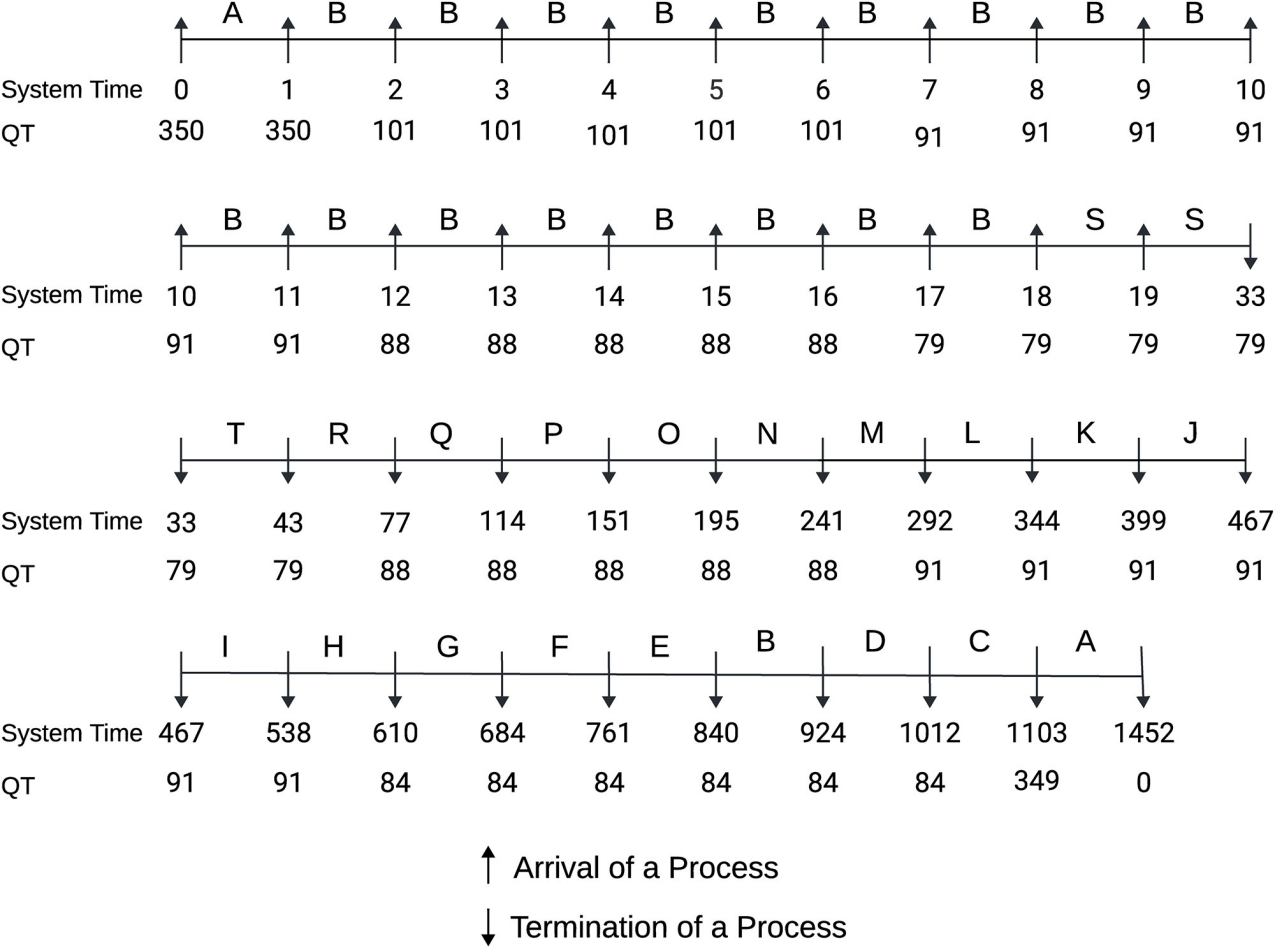
Gantt chart of the process with burst time in descending order.

Here, all the processes are assumed to arrive at the system with their burst time in a decreasing manner. Fig 4 depicted the Gantt chart of the process execution with determined QT at every checkpoint. At time 0, process A arrived in the RQ with BT = 350 and was selected by the scheduler for execution with QT = 350. Then, at time 1, process B arrived with BT = 101 and updated the QT as the 80th percentile BT of available processes, which is 350, and preempted process A selecting process B for execution as the BT of process B (101) is less than one-third of the current QT(117). Through this approach, we can eradicate the convoy effect that causes a small process to suffer for a comparatively large process. Process B is kept running till time 18, while we have 19 available processes (A, B, C, D, E, F, G, H, I, J, K, L, M, N, O, P, Q, R, and S) in our RQ. The algorithm determined QT = 79 at time 18 preempted Process B and shifted CPU to Process S because Process S had BT = 15, which is less than one-third of the current QT of 27. At time 19, a new process T entered the RQ with BT = 10, updating the QT = 79. We kept process S running till its completion as the remaining BT of S is smaller than one-third of the current QT prohibiting unnecessary context switching. In this way, the scheduler kept updating the QT based on the remaining BT of ready processes and preempted the running process or let it complete its execution accordingly. The proposed ERRDTQ completed all 20 tasks with 21 context switches, 431.90 AWT and 504.50 ATT.

#### Case 3: Burst time in random order

**Fig 5:**
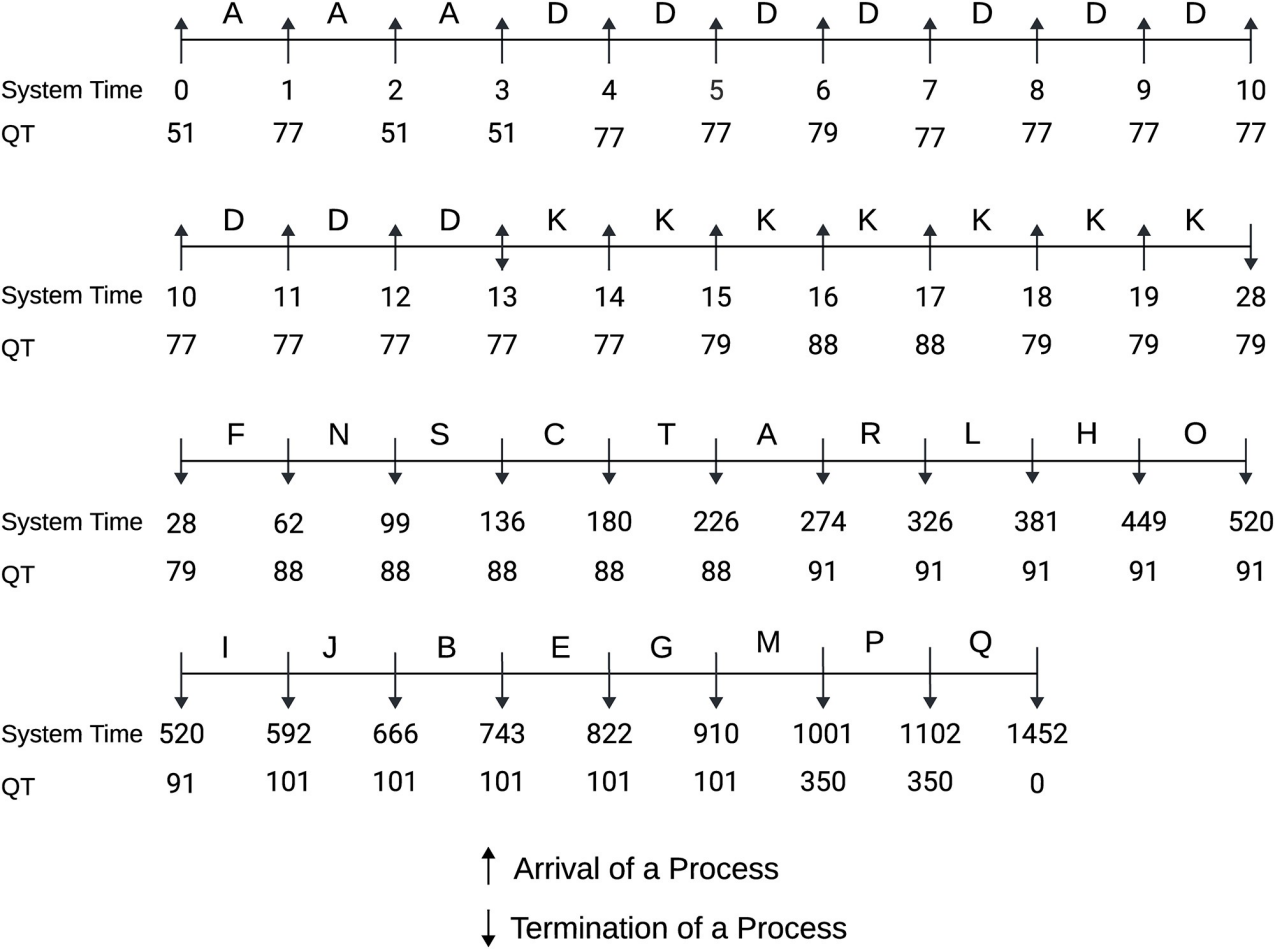
Gantt chart of process with burst time in random order.

Here, all the processes are assumed to arrive at the system with their burst time in a random order. Fig 5 depicted the Gantt chart of the process execution with determined QT at every checkpoint. At time 0, process A arrived in the RQ with BT = 51 and started its execution on the CPU with QT = 51. The algorithm updated QT to 77 with the arrival of process B (BT = 77) at time 1. We kept running process A because process B could not fulfil the preemption criteria. Then, at time 3, we have four processes (A, B, C, and D) in the SRQ and derived QT = 51. The scheduler preempted process A and transferred CPU to process D as it had BT = 10, which is smaller than one-third of the current QT (17). Process D terminated and left the system at time 13 when there were 13 (A, B, C, E, F, G, H, I, J, K, L, M, N) processes in the SRQ. Among them, the scheduler selected process K for execution with QT = 77. Within the completion of process K, we got all the other processes in the system and continued process execution without any further preemption until time 1452. The proposed ERRDTQ completed all 20 tasks with 19 context switches, 416.95 AWT and 489.55 ATT.

### Result comparison and discussion

To evaluate the effectiveness of the proposed algorithm, we compared this with five other improved round-robin approaches. The comparison results are presented in Tables 5–7 for process with their burst time in ascending order (Table 2), descending order (Table 3) and random order (Table 4) respectively. Each table portrays the performance of different models considering four evaluation matrics: Time Quantum (the duration for which each process is allowed to run), AWT (the amount of time processes spend in the ready queue), ATT (the amount of time processes take to complete their tasks), and the number of context switches (the number of times each model made processes swap in and out). The comparison shows that the proposed ERRDTQ outperforms other popular round-robin task scheduling methods by reducing AWT, ATT, and the number of context switches. In Table 5, it is depicted that the ERRDTQ algorithm performed well with the smallest AWT, ATT, and number of CS. The NRRTSA also showed the same performance for this case because shorter-length processes arrived prior in the system which automatically prevented the convoy effect (a process with a short burst time is stuck waiting for a process with a long burst time to complete).

**Table 5:** Comparative result of processes with burst time in ascending order.

Different Algorithms	SIDRR (Omotehinwa et al., 2019 [21])	DRRHA (Alhaidari et al., 2021 [26])	MARR (Sakshi et al., 2022 [33])	NRRTSA (Dipto et al., 2023 [38])	ARRA (Nermeen et al., 2023 [42])	ERRDTQ (This study)
Time Quantum	77, 350	5.5, 40.5, 39, 38, 48, 47, 133	10, 45, 74, 37	10, 67, 349	8, 33, 63	[Fig 3]
Average Waiting Time	415.95	444.45	493.95	415.95	429	415.95
Average Turnaround Time	488.55	517.05	566.55	488.55	501.6	488.55
No. of Context Switch	23	26	31	19	25	19

**Table 6:** Comparative result of processes with burst time in descending order.

Different Algorithms	SIDRR (Omotehinwa et al., 2019 [21])	DRRHA (Alhaidari et al., 2021 [26])	MARR (Sakshi et al., 2022 [33])	NRRTSA (Dipto et al., 2023 [38])	ARRA (Nermeen et al., 2023 [42])	ERRDTQ (This study)
Time Quantum	77, 350	175.5, 29, 30, 31, 32, 26, 19, 20, 14	350, 57, 22, 13	350, 100	263, 44, 27, 17	[Fig 4]
Average Waiting Time	457.75	749.45	799.25	693.35	776.6	431.90
Average Turnaround Time	530.35	822.05	871.85	765.95	849.2	504.5
No. of Context Switch	24	32	31	20	28	21

**Table 7:** Comparative result of processes with burst time in random order.

Different Algorithms	SIDRR (Omotehinwa et al., 2019 [21])	DRRHA (Alhaidari et al., 2021 [26])	MARR (Sakshi et al., 2022 [33])	NRRTSA (Dipto et al., 2023 [38])	ARRA (Nermeen et al., 2023 [42])	ERRDTQ (This study)
Time Quantum	77,350	26, 37, 38, 39,41, 47, 48, 134	51, 71, 30, 249	51,349	38,53	[Fig 5]
Average Waiting Time	422.65	450.4	522.05	422.65	438.4	416.95
Average Turnaround Time	495.25	523	594.65	495.25	511	489.55
No. of Context Switch	23	26	28	19	29	19

Table 6 shows that the ERRDTQ algorithm performed better than any other approach under consideration. Here, the process with the largest BT arrived in the system first and started execution while many smaller processes kept waiting in the ready queue. As our proposed algorithm updates its QT each time a new process arrives, it could make the best decision for preempting a running process and context switch. Through this technique the ERRDTQ achieved the best AWT and ATT for processes with variable length burst time automatically preventing the outliers. For this dataset, the NRRTSA recorded the best number of CS(20), whereas our approach scored with the number of CS(21), resulting in an improved AWT.

Based on the data presented in Table 7, we can observe that the ERRDTQ algorithm outperformed the others concerning AWT, ATT, and the number of CS. In this particular scenario, the algorithm processed incoming processes with varying burst times in a random order. By selecting the most appropriate quantum time (QT), the algorithm was able to minimize both the AWT and ATT while also ensuring that unnecessary context switches were avoided.

**Fig 6:**
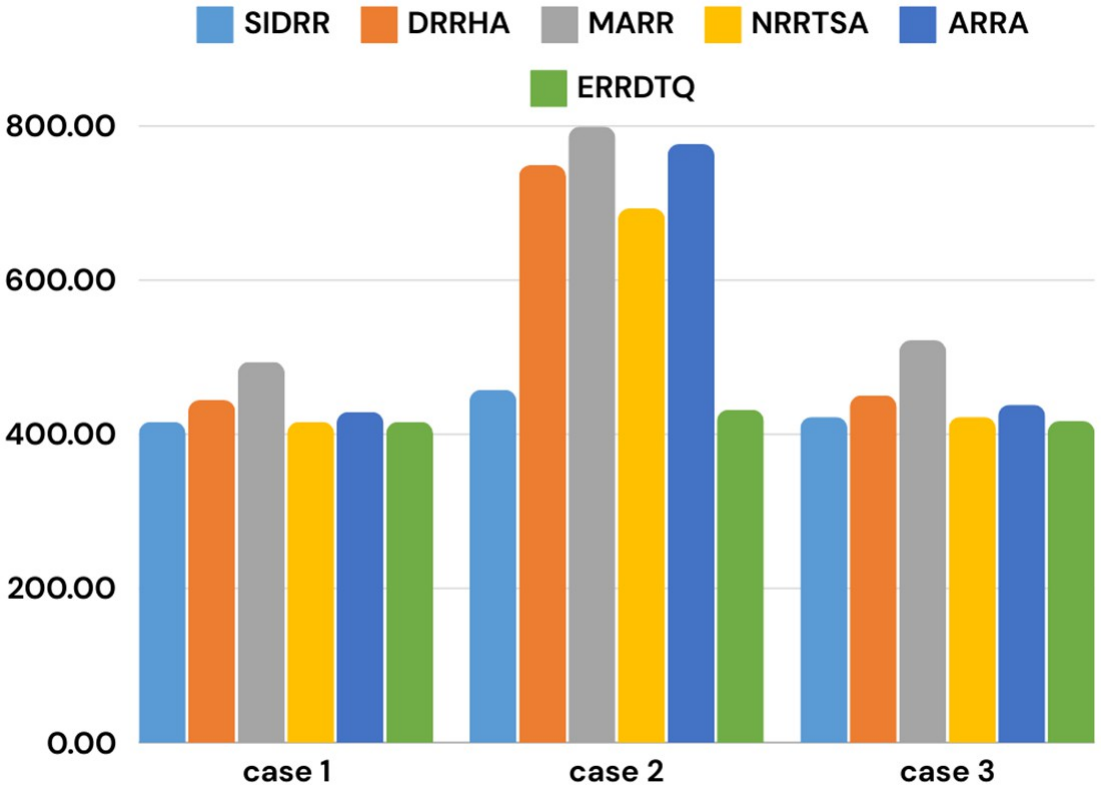
Comparison of AWT of different improved RR algorithms.

**Fig 7:**
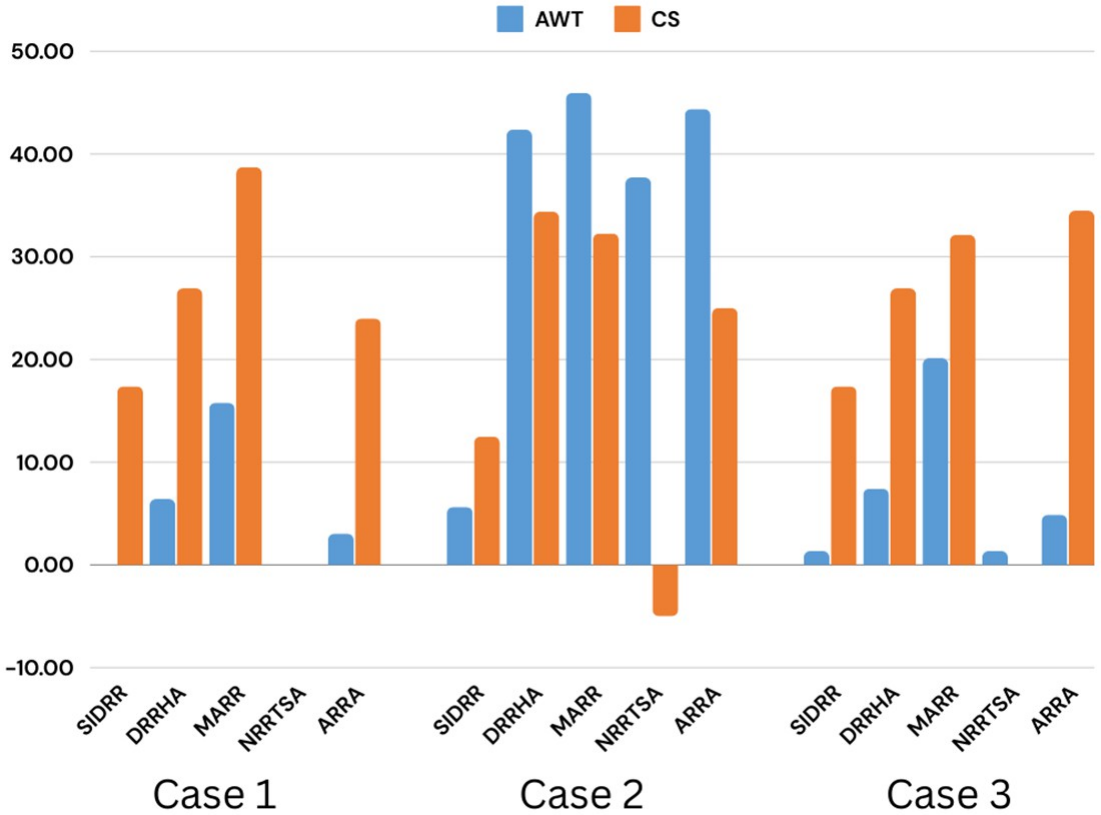
Improvement of ERRDTQ compared to the other related algorithms of round robin based on Average Waiting Time (AWT) and Context Switching(CS).

Fig 6 presents the AWT of the six improved RR approaches including ERRDTQ. It is depicted that for each case we scored the best result. In Fig 7, the proposed algorithm’s improvement rate is displayed. The algorithm resulted in a 5.05%, 35.22%, and 7.03% reduction in the average waiting time for case 1, case 2, and case 3, respectively. Moreover, it recorded a 20.03%, 19.83%, and 22.19% decrease in the number of context switching for the mentioned three cases individually.

As a consequence, it is clear that the proposed approach is a cost-effective, efficient, and feasible method of task scheduling for allocating resources in a cloud computing environment. It prioritizes relatively smaller tasks to decrease the convoy effect and ensures equitable CPU time distribution while avoiding unnecessary switching overhead.

## Conclusion and future direction

To maximize the utilization of resources, load balancing, reduce consumption of energy, adhere to service level agreements, and improve productivity and cost-effectiveness, optimal task scheduling is crucial in cloud computing. This paper presents a novel enhanced round-robin task scheduling method utilizing dynamic time quantum to improve the performance of task scheduling in a cloud computing environment. In addition, by automatically scheduling tasks of varying lengths and anticipating outlier burst times, the implemented ERRDTQ approach can provide minimal waiting time and a fair distribution of context switches. The results show that the suggested method performs better than the other round-robin task scheduling approaches currently in use in terms of decreasing the average waiting time, average turnaround time, and number of context switches. The study leads to the conclusion that the proposed ERRDTQ algorithm can be used in CC environment for scheduling processes with variable length BT with comparatively minimized operational cost. Our work did not incorporated the priorities of the incoming processes while planning the schedule. Our goal is to enhance the ERRDTQ methodology by taking into account the processes’ priorities and balancing them with high-quality performance through the application of reinforcement learning.
